# What did Sun Yat-sen really die of? A re-assessment of his illness and the cause of his death

**DOI:** 10.1186/s40880-016-0144-9

**Published:** 2016-09-02

**Authors:** Rolf F. Barth, Jie Chen

**Affiliations:** 1 Department of Pathology, The Ohio State University, Columbus, OH 43210 USA; 2 Department of Pathology, Peking Union Medical College Hospital, Beijing, 100730 P. R. China

**Keywords:** Sun Yat-sen, Terminal illness, Autopsy, Cause of death, Adenocarcinoma of the gallbladder

## Abstract

This year is the 150th anniversary of the birth of Sun Yat-sen (November 12, 1866) and the 91st year following his death (March 12, 1925). It generally has been believed that the cause of his death was “liver cancer.” However, as indicated in the official autopsy report, dated March 13, 1925, of the Peking Union Medical College Hospital (PUMCH) in Beijing, the cause of his death in reality was an adenocarcinoma of the gallbladder with direct extension to the liver and diaphragm as well as widespread metastases to the peritoneal cavity. This important piece of information seems to have never been reported in the English language literature, and it was only in 2013 that the true cause of his death was stated in a one-line sentence in a non-medical Chinese online source. It had been mistakenly believed that the cause of Dr. Sun’s death was liver cancer, based on the observations made following an exploratory laparotomy, which had been performed at PUMCH on January 26, 1925. The purpose of this short report is to provide more details relating to his terminal illness and to correct the historical record for a medical audience as to the cause of the death of Sun Yat-sen, a very important figure in the history of 20th century China.

## Background

**Fig. 1:**
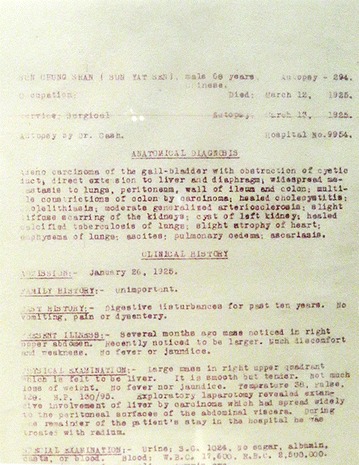
A barely legible copy of the autopsy report on Sun Yat-sen that is on display in the Sun Yat-sen Memorial Hall

**Fig. 2:**
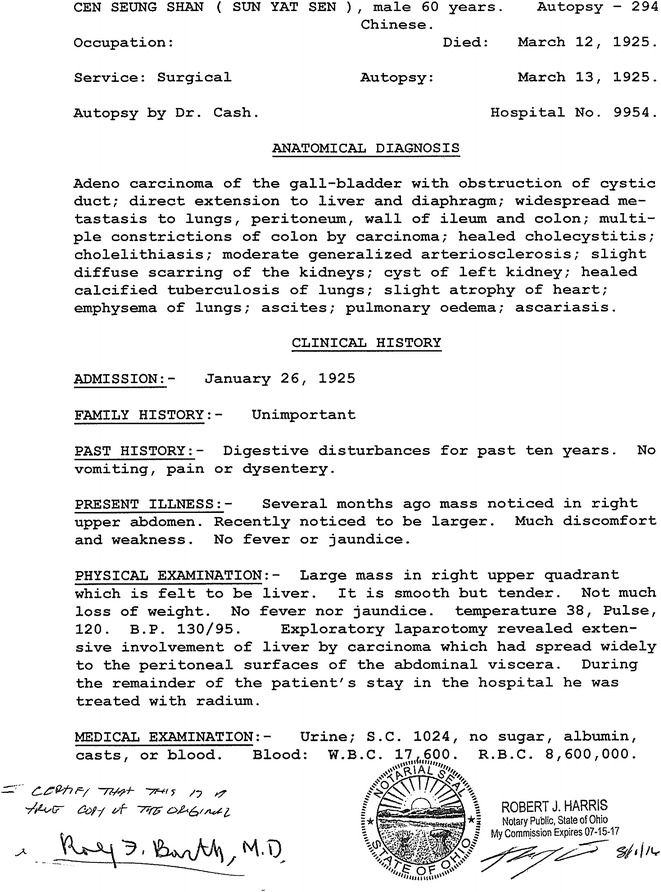
A clearly legible retyped, notarized copy of the original Autopsy Report on Dr. Sun

One of us (RFB) visited the Sun Yat-sen Memorial Hall in Guangzhou, China in early May 2016. Among the many items in the exhibition hall display cases relating to the life and accomplishments of Dr. Sun was a barely legible, one-page document that was of special interest to us as pathologists. It was a poor-quality, carbon copy of the autopsy report on Sun Yat-sen (Fig. 1), dated March 13, 1925, who had died at 9:00–9:30 a.m. on March 12, 1925 in Beijing. A limited autopsy was performed at the PUMCH by James R. Cash, MD, an associate professor in the Department of Pathology. This report, written in English (Fig. 2), indicated that the anatomic diagnoses were as follows:

“adenocarcinoma of the gallbladder with obstruction of the cystic duct; direct extension to liver and diaphragm; widespread metastasis to lungs, peritoneum, wall of ileum and colon; multiple constrictions of colon by carcinoma; healed cholecystitis; cholelithiasis; moderate generalized arteriosclerosis; slight diffuse scarring of the kidneys; cyst of left kidney; healed calcified tuberculosis of lungs; slight atrophy of heart; emphysema of lungs; ascites; pulmonary oedema; ascariosis [*sic* ascariasis].”

**Fig. 3:**
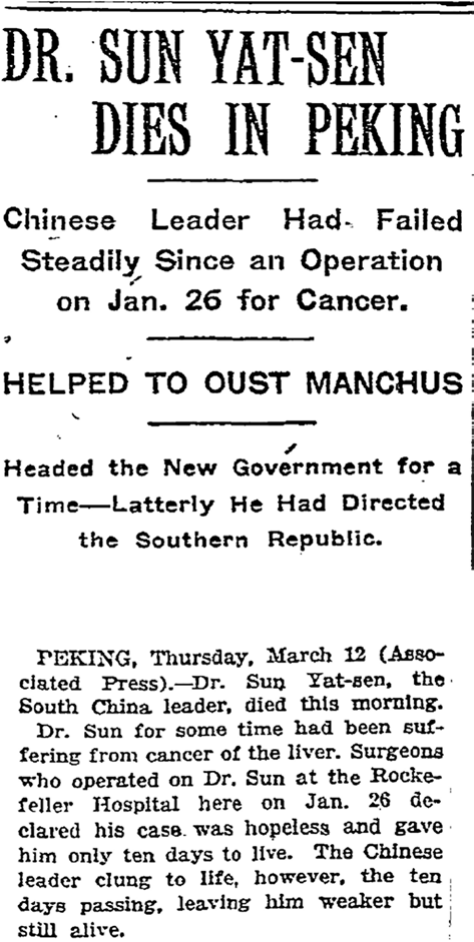
Excerpt from Dr. Sun Yat-sen’s obituary from *The New York Times* dated March 12, 1925 Copyright © *The New York Times*

The diagnosis of adenocarcinoma of the gallbladder was completely unexpected by us, since it had been widely reported in obituaries such as those in *The New York Times* dated March 12, 1925 (Fig. 3) [1], *TIME* magazine dated March 23, 1925 [2], and the Chinese newspaper *Qun Qiang Bao* dated March 15, 1925 [3], that “Dr. Sun had for some time been suffering from cancer of the liver.” It was further stated in *The New York Times* article [1] that surgeons operated on Dr. Sun at the “Rockefeller Hospital,” or as it also was known, PUMCH, on January 26, 1925, and it was declared that his case was hopeless and he only had 10 days to live. However, he was still alive after 10 days, although weaker.

## Clinical history

Assuming that they still exist, the original Autopsy Report and medical records relating to Dr. Sun’s illness could not be located at the time of the writing of this report. On the other hand, the Seventeenth Annual Report for the year of 1925 of PUMCH has survived [4], but it sheds no light on the clinical history and autopsy of Dr. Sun. In the brief clinical history of Dr. Sun’s illness drafted by Dr. Cash (Figs. 1 and 2), it was stated “that several months earlier a mass had been noted in the right upper abdomen, and that shortly before he was operated on it was noted to be larger and causing him much discomfort and weakness. Physical examination prior to his surgery revealed a large mass in the right upper quadrant of his abdomen and this was thought to be his liver. It is smooth but tender. Not much loss of weight. No fever or jaundice. Temperature 38°, Pulse, 120, BP 130/95.”

The exploratory laparotomy was carried out on January 26, 1925, by Dr. Adrian S. Taylor, Head of the Department of Surgery at PUMCH [4, 5]. It “revealed extensive involvement of the liver by carcinoma that had spread widely to the peritoneal surfaces of the abdominal viscera.” As indicated in the autopsy report, during the remainder of his hospital stay he was “treated with radium,” although no details were provided. However, as reported in *The New York Times* [1], on February 18, 1925, “against the advice of hospital authorities, Dr. Sun was removed by friends and political associates to the headquarters of the Kuomintang, the former residence of Wellington Koo, previous Foreign Minister.” Dr. Sun also acceded to the entreaties of those close to him and was treated by “old style Chinese doctors” [5], but his clinical condition continued to deteriorate. It was further noted in *The New York Times* article [1] that, “following his transfer from PUMCH he was growing weaker and weaker over the next few weeks, and shortly before his death he refused to accept food.” Following his death on March 12, 1925, his body was placed in a wooden coffin, covered with a blanket [3], and transported from the Koo residence to PUMCH, where a limited autopsy was performed by Dr. Cash on March 13, 1925 “under the watchful gaze of his son, Sun Fo, and an entourage of military generals” [6]. Following this, “in the face of considerable opposition, the family had the courage to arrange for a Christian funeral service that was held in the beautiful auditorium of the PUMCH” [5].

## Pathologic examination

**Fig. 4:**
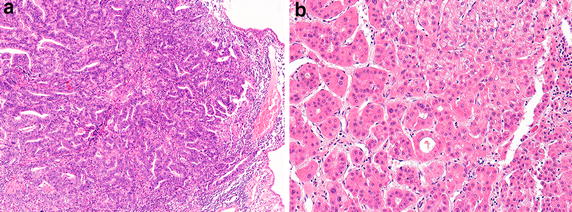
Representative photomicrographs of **a** an adenocarcinoma of the gallbladder and **b** a hepatocellular carcinoma (magnification ×100). The fact that Dr. Sun’s tumor was highly invasive of the liver and had metastasized to a number of different organs suggests that it could have been a poorly differentiated adenocarcinoma [9]

According to a brief description of the autopsy itself [7], only a small section of tissue was taken for microscopic examination since the family had stipulated that no organs were to be removed from the body [6]. Subsequently, as described by Choa [8], during the Japanese occupation of Beijing, the military authorities announced that the internal organs, of which in reality there were none since they had never been removed from the body, and the microscopic section of the tumor, which had been kept in the Department of Pathology at PUMCH, would be reunited with Dr. Sun’s body in the mausoleum in Nanjing, which had been completed in May 1929. According to Bowers and Choa [7, 8], this single microscope slide was sent to Nanjing in an enormous chest, and presumably it was placed in Dr. Sun’s sarcophagus. The Japanese hoped that by this gesture they could boast that they had righted the wrong done to the Chinese national hero by foreigners, i.e., the Americans [7]. Consequently, we cannot show any photomicrographs of his tumor, but we have selected representative photomicrographs of an adenocarcinoma of the gallbladder (Fig. 4a) and a hepatocellular carcinoma (Fig. 4b) to illustrate the histopathologic differences between the two malignant tumors (Figs. 1 and 2). As indicated in the autopsy report (Fig. 2), the adenocarcinoma had infiltrated the liver and metastasized very widely both in the peritoneal cavity and to the thoracic cavity involving the lungs.

This pattern of metastasis was entirely consistent with a diagnosis of adenocarcinoma of the gallbladder [9, 10]. In contrast, “liver” cancer, and to be more specific, hepatocellular carcinoma, has a completely different pattern of metastasis [11], which most frequently begins with invasion of the portal vein and from there to the lymph nodes (stage III disease), and then more distant anatomic sites including the lungs (stage IVb). As summarized by Lazcano-Ponce et al. [10], over 90% of gallbladder carcinomas are adenocarcinomas. Gallstones and evidence of chronic cystitis are found in almost all cases, as was the case with Dr. Sun, and most of these are well to moderately differentiated adenocarcinomas, although in the absence of the histologic sections of Dr. Sun’s tumor, we cannot make any definitive statement regarding this. However, based on its aggressive pattern of growth, it could have been a poorly differentiated adenocarcinoma. Sadly, even today, patients with stage IVb gallbladder cancer have a very poor prognosis with a 5-year survival rate of only 2% [12].

## Conclusions

In summary, what is remarkable to us is that it has taken 91 years after Dr. Sun’s death to correct the historical record in the English language literature and only recently in a non-medical Chinese online source [13] regarding the cause of his death. Purely by chance, it was an American pathologist visiting the Sun Yat-sen Memorial in Guangzhou in early May 2016 who noticed a barely legible, one-page document and examined it closely enough to see that it was a copy of the autopsy report of Dr. Sun, and it indicated that he had succumbed to an adenocarcinoma of the gallbladder and *not* liver cancer. Why then was it reported on March 12, 1925 in *The New York Times* [1], *TIME* magazine [2], and a Chinese newspaper [3] that Dr. Sun had died of liver cancer? Most probably, it was based on the observations of Dr. Taylor, who had performed the exploratory laparotomy and found that Dr. Sun’s liver had been massively infiltrated with a malignant tumor. It was very reasonable, therefore, for him to conclude that Dr. Sun had liver cancer. However, following the autopsy on Dr. Sun, it was definitively established by Dr. Cash’s histopathologic examination that the correct diagnosis was adenocarcinoma of the gallbladder, which grossly had been noted to be stone hard [3]. The incidence of liver cancer in present day China is 7.6 times greater than that of gallbladder cancer [14]. Since cancer statistics for China in 1925 were not available, we can only speculate on what the difference in incidences would have been at that time, but most certainly liver cancer was more common than gallbladder cancer, and hence Dr. Taylor’s conclusion. If the information in Dr. Cash’s autopsy report had been transmitted to the press back in March 1925, there would have been no need to correct the historical record. However, to the best of our knowledge, it was not. The final take-home message is that, even today, in the age of molecular medicine with a variety of sophisticated diagnostic techniques, a carefully performed autopsy still can provide answers to important questions that otherwise would remain unanswered.

## Abbreviation

PUMCH: Peking Union Medical College Hospital
